# Levetiracetam versus Oxcarbazepine as monotherapy in newly diagnosed focal epilepsy: A systematic review and meta‐analysis

**DOI:** 10.1002/brb3.2779

**Published:** 2022-10-02

**Authors:** Sanjeev Kharel, Rajeev Ojha, Surendra Khanal

**Affiliations:** ^1^ Maharajgunj Medical Campus Tribhuvan University Institute of Medicine, Maharajgunj Kathmandu Nepal; ^2^ Department of Neurology Tribhuvan University Teaching Hospital, Maharajgunj Kathmandu Nepal

**Keywords:** focal epilepsy, Levetiracetam, monotherapy, Oxcarbazepine

## Abstract

**Objective:**

To compare the efficacy and safety of Levetiracetam (LEV) and Oxcarbazepine (OXC) as monotherapy for the treatment of newly diagnosed focal epilepsy.

**Methods:**

We searched PubMed, Cochrane Library, EMBASE, and Google Scholar from January 1, 2000 to May 11, 2022, with no language restrictions along with The ClinicalTrials.gov website and the WHO International Controlled Trials Registry platforms. We pooled the risk ratio (RR) and corresponding 95% confidence interval (95% CI) for the efficacy and safety outcomes. The quality of included trials was assessed using the Cochrane Collaboration's tool.

**Results:**

Two RCTs included a total of 574 newly diagnosed focal epilepsy patients (the LEV group [282 patients] and the OXC group [292 patients]). LEV group when compared with the OXC group had no significant difference in the pooled estimate of seizure freedom at week 24. (RR: 0.81; 95% CI: 0.62–1.05, *p* = .11). Similarly, there was no significant difference in the pooled estimate of withdrawal due to adverse events (AEs) (RR: 0.87; 95% CI: 0.34–2.23, *p* = .77). The commonly reported AEs in both trials were dizziness, headache, rash, somnolence, and nasopharyngitis with zero medication‐related death and few serious AEs.

**Conclusions:**

LEV is noninferior to OXC in terms of seizure freedom at week 24 and treatment withdrawal rate due to AEs among adults but long‐term treatment data is still missing. Future multicentric double‐blinded RCTs and real‐world studies are of great need.

## Graphical abstract

LEV is non‐inferior to OXC in terms of seizure freedom at 24‐weeks and treatment withdrawal rate due to AEs among adults but long‐term treatment data is still missing.

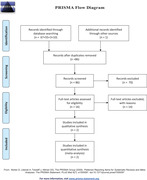

## 1 INTRODUCTION

Epilepsy is the fourth‐leading neurological disease in the world affecting around 50 million in the world. The annual incidence of epilepsy is about 80 cases per 100,000 individuals (Epilepsy Foundation 2019; World Health Organization, 2019). The International League Against Epilepsy has classified epilepsy as focal, generalized, combined, and unknown (Fisher et al., 2017). Focal epilepsy, the most common type occurs within the area limited to a single hemisphere (Fisher et al., 2017). The main target of antiseizure medications (ASMs) treatment is to achieve complete seizure freedom without inducing adverse events, to decrease mortality and morbidity, and to improve the patient's quality of life (Sander, 2005).

Levetiracetam (LEV) and Oxcarbazepine (OXC) are the commonly used ASMs for the focal epilepsy (Kanner et al., 2018; Shorvon, 2000). OXC is a keto analogue of the drug carbamazepine (CBZ) so similar to mechanism of action CBZ (Garoufi et al., 2016). LEV on other hand is a broad‐spectrum ASM that works by modulation of neurotransmission through vesicle protein 2A (Grinspan et al., 2018). In comparison to other many ASMs, both LEV and OXC are highly effective in controlling seizures (Howard et al., 2018).

Few studies have compared the safety and efficacy of these drugs as a monotherapy for epilepsy (Kim et al., 2017; Li et al., 2020; Zhu et al., 2022). Thus, we aim to systematically review and perform a meta‐analysis to compare the effectiveness and safety of LEV and OXC in the treatment of newly diagnosed focal epilepsy.

## 2 MATERIALS AND METHODS

This systematic review and meta‐analysis was performed and reported in compliance with the Preferred Reporting Items for Systematic Reviews and Meta‐Analyses (PRISMA) Statement (Moher et al., 2009) with a predefined review and data extraction protocol. When compared to a single study, a meta‐analysis of published trials can increase the statistical power (Lee, 2018). Thus, the two trials conducted to compare the effectiveness and safety evidence of LEV and OXC need further investigations as these provide contrasting findings. The efficacy outcomes of this study were seizure‐freedom rate and treatment failure rate. Safety outcome will be assessed by using variables such as number and percentage of patients with AEs, discontinuation rate or dropout rate. The discontinuation frequency was calculated for any of the following three reasons: an adverse event (AE) related to the trial drug, lack of efficacy, or the need for an additional AED. The discontinuation rate and dropout rate as a whole were combined in one outcome i.e., treatment failure rate. Serious adverse events are those that result in death, are life‐threatening, and require hospitalization or prolongation of existing hospitalization.

### 2.1 Inclusion and exclusion criteria

#### 2.1.1 Inclusion criteria

Studies must be blinded randomized controlled trials comparing the efficacy and safety of Levetiracetam or Oxcarbazepine as monotherapy.

**Participants**: All of the adult subjects with newly diagnosed focal epilepsy.

**Interventions**: Newly diagnosed focal epilepsy treated with Levetiracetam or Oxcarbazepine as a monotherapy.

**Comparator**: Efficacy and safety of two antiepileptic drugs (LEV and OXC).

**Outcomes**: The outcomes include: Seizure‐freedom rate, treatment failure rate, AEs and AEs‐related withdrawal rate.

#### 2.1.2 Exclusion criteria

Studies were excluded if they met any criteria as follows: (a) treatment of diseases other than new onset focal epilepsy, (b) observational, retrospective studies or trials with less than 20 participants, and reviews, letters, editorials, conference abstracts, and meta‐analyses, (c) the detailed data on efficacy and safety profiles were not available.

### 2.2 Search strategy and selection

The databases searched were PubMed, Cochrane Library, EMBASE, and Google Scholar from January 1, 2000 to May 11, 2022, with no language restrictions. The ClinicalTrials.gov website and the WHO International Controlled Trials Registry platforms also were retrieved for ongoing and completed studies reporting results. Our search terms included “levetiracetam,” “oxcarbazepine,” “focal epilepsy,” “partial seizure,” and “Focal seizure.” The detailed search strategy is given in supplementary file 1. Additionally, references included in eligible research and reviews were checked to see whether any additional studies met our eligibility requirements.

Two independent reviewers (SK and RO) searched the databases and screened the articles according to the inclusion and exclusion criteria. Any conflicts were resolved by a third author. (SK’)

### 2.3 Data extraction and quality assessment

We extracted the following data in an Excel spreadsheet from each RCT with a predefined form consisting of the author, study type, study site, number of patients, mean/median age, female number, duration of epilepsy, and doses used for LEV and OXC groups, efficacy outcomes and safety outcomes of LEV and OXC groups.

The Cochrane risk of bias tool was used to evaluate the quality of the RCTs. It includes seven items: random sequence generation, allocation concealment, blinding of participants and personnel, blinding of outcome assessment, incomplete outcome data, selective reporting, and other biases. Each item was divided into low‐risk, unknown, and high‐risk (Higgins et al., 2011).

### 2.4 Data synthesis and analysis

We pooled the risk ratio (RR) and corresponding 95% confidence interval (95% CI) for the efficacy and safety outcomes. Heterogeneity between the included studies was determined using the *I* ^2^ test (Higgins, 2003). The presence of *I* ^2^ > 50% was considered an indicator of significant heterogeneity. The Mantel–Haenszel method was used for analyses (Statistical Aspects of the Analysis of Data From Retrospective Studies of Disease, 1959). Forest plots with 95% CIs were created to show individual study results and weights as well as overall pooled estimates. A *p* value of < .05 was considered statistically significant. Statistical analysis was performed using Revman v.5.3 (Nordic Cochrane Centre, Copenhagen, Denmark).

## 3 RESULTS

### 3.1 Literature search

**FIGURE 1:**
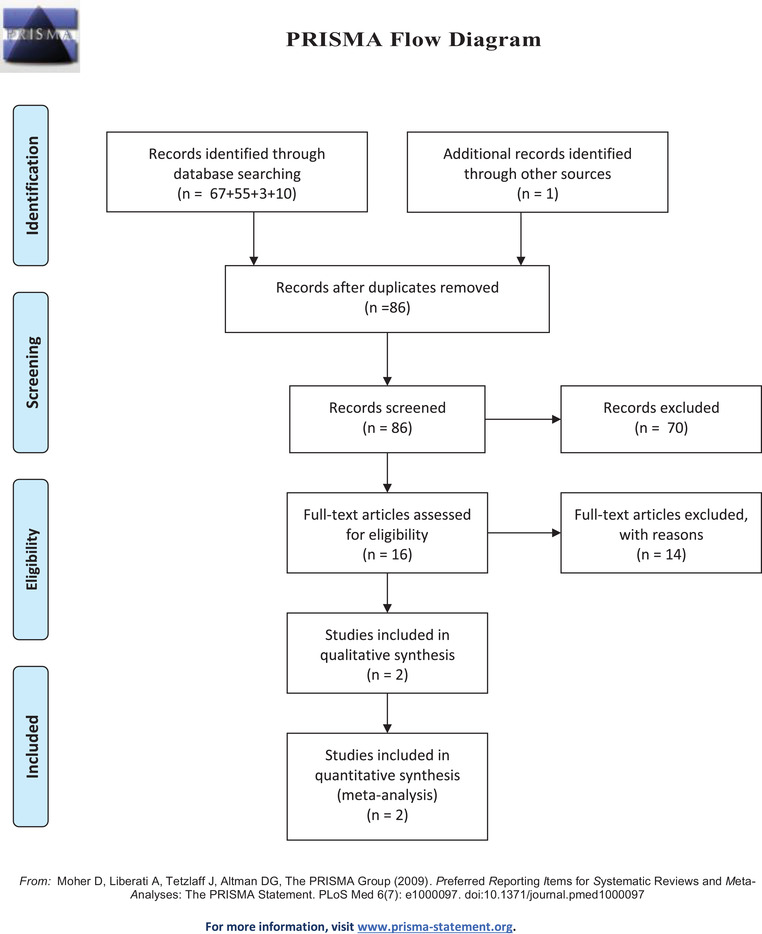
The PRISMA flowchart showing the study selection and inclusion process.

The database search yielded 136 articles initially, of which 50 were excluded for duplications. Out of the remaining 86 articles, 70 were excluded after screening the titles and abstracts based on eligibility criteria. Full‐text reviews were performed among the 16 articles and finally, 2 RCTs were included in this meta‐analysis (Kim et al., 2017; Zhu et al., 2022). The PRISMA flowchart (Figure 1) shows the study selection and inclusion process.

### 3.2 Study characteristics

The detailed demographic and study characteristics are shown in Table 1. Two RCTs included in our study were open‐label studies published in 2017 and 2022 (Kim et al., 2017; Zhu et al., 2022). In both trials, enrolled participants were older than 18 years with newly diagnosed focal epilepsy according to the International League against Epilepsy's Classification of Epileptic Seizures (Fisher et al., 2017).

**TABLE 1:** Study characteristics and patients details in each intervention group (LEV and OXC)

				Intervention group										Efficacy outcome		Safety outcome	
				LEV group					OXC group								
Author	Study type	Site	Patients included	No. of patients	Mean/median age (years)	Female *n*, (%)	Duration of epilepsy (months)	Doses	No. of patients	Mean/Median age (years)	Female, *n* (%)	Duration of epilepsy (months)	Doses	LEV group	OXC group	LEV group	OXC group
Kim 2017	RCT (NCT01498822)	South Korea	Korean patients (16–80 years), with ≥2 unprovoked focal seizures in the year preceding the trial, who had not taken any antiepileptic drugs (AEDs) in the last 6 months, were randomized to receive LEV or OXC (1:1)	173	39.5 (16.6)	83 (48%)	1.85 (9.41)	1000 mg/day, uptriated maximum dose: 3000 mg/day	174	42.2 (17.2) years	76 (43.7%)	0.92 (1.46)	900 mg/day, uptriated maximum dose: 2400 mg/day	Seizure freedom rate: (at week 24: 93/173 [53.8%], at week 48: 60/173 [34.7%]); treatment failure rate: 19/173 (11%)	Seizure freedom rate: (at week 24: 100/171 [58.5%], at week 48: 70/171 [40.9%]); treatment failure rate: 31/171 (18.1%)	Discontinuation due to TEAEs: 11/173 (6.4%), serious TEAE: 15/173 (8.7%), TEAEs with dose change: 10/173 (5.8%)	Discontinuation due to TEAEs: 20/174 (11.5%), serious TEAE: 15/174 (8.6%), TEAEs with dose change: 19/174 (10.9%)
Zhu 2022	RCT (ChiCTR‐OCH‐ 14004528)	China	Patients with newly diagnosed focal epilepsy who had experienced 2 or more unprovoked seizures at greater than a 24‐h interval during the previous year were recruited and randomly assigned to LEV or OXC group	109	32 (24–45)	55 (41.6%)	17 (4–66)	1000 mg/day, uptriated maximum dose: 3000 mg/day	118	37 (26–51) years	53 (49.1%)	10.5 (3–50.25)	900 mg/day, uptriated maximum dose: 2400 mg/day	Seizure freedom rate: (at week 12: 62/100 [62%], at week 24: 48/90 [53.3%]); no response or worsening: (at week 12: 31/100 [31%], at week 24: 19/90 [21.1%])	Seizure freedom rate: (at week 12: 91/113 [80.5%], at week 24: 82/108 [75.9%]); no response or worsening: (at week 12: 14/113 [12.4%], at week 24: 6/108 [5.6%])	Dropout due to AEs: 12/109 (11%), adverse events: 4/90 (4.4%)	Dropout due to AEs: 9/118 (7.6%), adverse events: 5/108 (4.6%)

Two RCTs included a total of 574 newly diagnosed focal epilepsy patients. There were two intervention groups for the treatment: the LEV group (282 patients) and the OXC group (292 patients). Both studies were multicenter RCTs conducted in South Korea and China. Both studies had the same first dosage level of 1000 mg/day for LEV and 900 mg/day for OXC and if seizures were not controlled during the treatment period, dosages were up titrated up to 3000 mg/day and 2400 mg/day for LEV and OXC, respectively. In both trials, males were predominance in number in both groups. Similarly, the mean duration of epilepsy before initiation of AEDs was greater in the LEV intervention group compared to the OXC intervention group in both trials (Kim et al., 2017, Zhu et al., 2022). Kim et al. (2017) had a 48‐week follow‐up period and Zhu et al. (2022) had only a 24‐week follow‐up period.

### 3.3 Efficacy outcome

#### 3.3.1 Seizure Freedom rate

**FIGURE 2:**
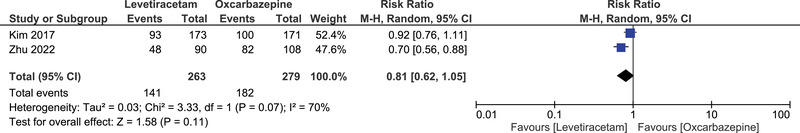
The forest plot depicting the pooled risk ratios of two RCTs for seizure freedom at week 24. The area of each square is proportional to the study's weight in the meta‐analysis, while the diamond shows the pooled result. The horizontal lines through the square illustrate the length of the confidence interval. The width of the diamond serves the same purpose. The overall meta‐analyzed measure of effect is an imaginary vertical line passing through the diamond

We conducted a meta‐analysis comparing LEV and OXC for only one efficacy outcome: Seizure Freedom rate at week 24 as the data on treatment failure was not uniform for the analysis. Patients treated with LEV (*n* = 263) with seizure freedom (*n* = 141) when compared with patients treated with OXC (*n* = 279) with seizure freedom (*n* = 182) had no significant difference in the pooled estimate (RR: 0.81; 95% CI: 0.62–1.05, *p* = .11). A random‐effect model was opted for high heterogeneity (*I* ^2^ = 70%) in our analysis. The forest plot depicting the pooled estimate of two RCTs is given in Figure 2.

#### 3.3.2 Safety outcome

**FIGURE 3:**
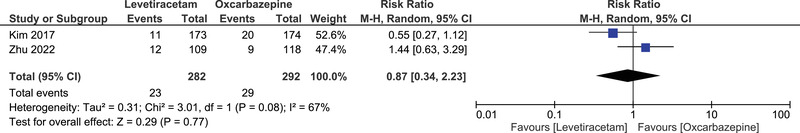
The forest plot depicting the pooled risk ratios of two RCTs for treatment withdrawal due to AEs. The area of each square is proportional to the study's weight in the meta‐analysis, while the diamond shows the pooled result. The horizontal lines through the square illustrate the length of the confidence interval. The width of the diamond serves the same purpose. The overall meta‐analyzed measure of effect is an imaginary vertical line passing through the diamond

For safety outcomes, we conducted a meta‐analysis comparing LEV and OXC under treatment withdrawal rate due to AEs. Patients treated with LEV (*n* = 282) with withdrawal (*n* = 23) when compared with patients treated with OXC (*n* = 292) with withdrawal (*n* = 29) had no significant difference in the pooled estimate (RR: 0.87; 95% CI: 0.34–2.23, *p* = .77). A random‐effect model was opted for high heterogeneity (*I* ^2^ = 67%) in our analysis. The Forest Plot depicting the pooled estimate of two RCTs is given in Figure 3. The commonly reported AEs in both trials were dizziness, headache, rash, somnolence, and nasopharyngitis. There were zero fatal cases in both trials due to study medication. Only a few serious AEs were observed in one trial (Kim et al., 2017) while others had no serious AEs (Zhu et al., 2022).

#### 3.3.3 Risk of bias

**FIGURE 4:**
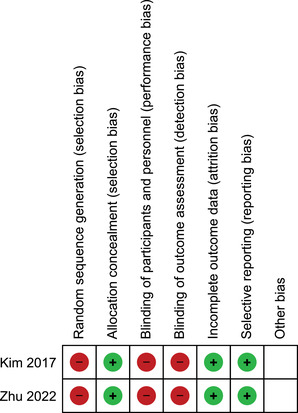
The risk of bias diagram of two RCTs using The Cochrane risk of bias tool

In both RCTs, selection bias, performance bias, and detection bias had high risk while the remaining other types of bias had low risk. The risk of bias diagram of two RCTs is depicted in Figure 4.

## 4 DISCUSSION

This study showed a statistically nonsignificant difference between the LEV and OXC groups in terms of seizure freedom at week 24 and treatment withdrawal rate due to adverse events. Additionally, common treatment‐related adverse events were also observed in the trial. A previous meta‐analysis including all the types of epilepsy patients found LEV was as effective as OXC in rates of seizure freedom after treatment of 6 months and 12 months (Yi et al., 2019).

This is the meta‐analysis of two RCTs of adult patients diagnosed with newly diagnosed focal epilepsy. The South Korean trial found that 24‐week seizure freedom rates were similar for LEV and OXC but the 48‐week seizure freedom rates were numerically lower for LEV than OXC. But all these findings were not statistically significant (Kim et al., 2017). While, in contrast, a trial in China found that efficacy in the OXC group had significantly higher number of seizure‐free patient at 12 and 24‐week than in the LEV group (Zhu et al., 2022). A observational study compared LEV, OXC, and Lamotrigine(LTG) and found that LEV had the greater 1‐year seizure‐free rate (65.7%) than LTG (60.2%) and OXC (53.4%) with no significant differences for 1 year seizure freedom rate between them (Li et al., 2020). The pooled data of these trails showed a nonsignificant finding. The explanation for the contrasting results between trials and real‐world studies may be due to patient age group variations, differences in study population, their baseline characteristics and different titration doses used.

The real‐world study found that the 3‐year seizure‐free rate for LEV (41.4%) was significantly better than OXC (26.2%) for newly diagnosed focal seizure (Li et al., 2020). While in study with a 3‐year follow‐up, the pediatric patients with epilepsy taking LEV had the seizure‐free rate progressively increasing over time. Seizure‐free rates increased over time, 13%, 15%, and 18% at 1, 2, and 3 years, respectively (Zhao et al., 2021). This finding suggests that for long‐term treatment of newly diagnosed focal epilepsy LEV might be more effective than OXC. In other words, OXC initially has an edge in terms of effectiveness, but notably when used as a long‐term monotherapy for epilepsy, OXC's persistence of efficacy eventually declines in comparison to LEV (Zhu et al., 2022). This could be because OXC is less effective and a greater percentage of patients are discontinuing treatments because of side effects (Li et al., 2020).

A meta‐analysis described the safety of LEV and OXC in focal epilepsy patients. The difference was not significant in terms of treatment withdrawal rate owing to AEs when used as monotherapy but when recommended as adjunctive treatment, OXC had the highest withdrawal rate and AE rate (Jeon et al., 2021). In our study, the results of safety assessments about the common adverse events like headache, dizziness, somnolence, fatigue, and nausea were similar to the previous meta‐analysis results (Jeon et al., 2021; Zhao et al., 2017). The real‐world clinical practice also found similar adverse events. Rashes followed by dizziness and abnormal hepatic function were the most common adverse events (Li et al., 2020).

The trial from South Korea found a numerically higher treatment failure rate in the OXC group (18.1%) when compared to the LEV group (11%) (Kim et al., 2017). In real‐world clinical practice also, LEV was well tolerated and had the best efficacy and safety over time but the age of onset of LEV group was the youngest among the three AEDs who are considered safer due to the lower incidence of rashes. The 1‐year withdrawal rate for OXC (35.1%) was numerically greater than those for LEV (26.3%). OXC displayed the greater proportion of treatment failure cases due to unacceptable adverse effects (8.9%), while LEV (2%) had the least likelihood of resulting in treatment failure (Li et al., 2020). But in contrast, the Chinese trial found a statistically significant higher no response or worsening rate after treatment in the LEV group (21.1%) than in the OXC group (5.6%) (Zhu et al., 2022). The above results can vary because of the differences in titration doses and maintaining doses of drugs used, differences in study age groups and patients with drug resistant.

The good thing during the 24‐week follow‐up evaluation period both LEV and OXC could improve the quality of life and anxiety conditions in patients with focal epilepsy (Zhu et al., 2022). The seizure‐free rate, the time to treatment withdrawal, and the long‐term treatment withdrawal rate are the most valuable indices for the practicability of ASMs (Mohanraj & Brodie, 2003).

Hence, before conducting future studies, the above indices and quality of life should be considered to get the best possible result.

## 5 LIMITATIONS

Our study had several limitations. At first, the role of the new drugs investigated in this study (OXC, and LEV) as monotherapy is uncertain because the results of strict regulatory trials (short‐lasting and only in adults) cannot be easily transferred to clinical practice. Moreover, only two RCTs were included in the current meta‐analysis, which lowers the applicability of this study. Hence, the results should be a cautious interpretation of results is necessary. Similarly, RCTs included were open‐label with a high risk of bias. Also, only Asian population were included in the RCTs. Lastly, the analysis for efficacy was only done for a 24‐week treatment period so long‐term efficacy still needs to be evaluated.

## 6 CONCLUSION

Our study concluded that LEV is noninferior to OXC in terms of seizure freedom at week 24 and treatment withdrawal rate due to AEs among adults. But long‐term efficacy and safety data are still unclear pointing out the need for multicenter, multiethnic double‐blind RCTs and real‐world observational studies comparing LEV and OXC for a longer treatment period.

### PEER REVIEW

The peer review history for this article is available at: https://publons.com/publon/10.1002/brb3.2779

## Supporting information

supplementary material

## Data Availability

The data are available on the request of corresponding author.
