# Supplementary material for: Levetiracetam versus Oxcarbazepine as monotherapy in newly diagnosed focal epilepsy: A systematic review and meta‐analysis
Source: Brain Behav. 2022 Oct 2;12(11):e2779. doi: 10.1002/brb3.2779 (PMC9660411; doi:10.1002/brb3.2779)
Supplement: Supplementary file 1 — supplementary material [file BRB3-12-e2779-s001.docx]

**Search strategy**

**For PubMed**

**#1:"Epilepsies, Partial"[Mesh] OR "Focal epilepsy"**

**#2: "Levetiracetam" OR "Oxcarbazepine"**

 Filters: **Randomized Controlled Trial, English, Humans, from 2000 – 2022**

**Search strategy:**

(("epilepsies, partial"[MeSH Terms] OR "Focal epilepsy"[All Fields]) AND ("Levetiracetam"[All Fields] OR "Oxcarbazepine"[All Fields])) AND ((randomizedcontrolledtrial[Filter]) AND (humans[Filter]) AND (english[Filter]) AND (2000:2022[pdat]))

**Articles: 67**

**Clinical trials.gov=3 articles**
